# Obstructive Hypertrophic Cardiomyopathy in a Symptomatic Professional Athlete Treated With Mavacamten

**DOI:** 10.1016/j.jaccas.2026.108253

**Published:** 2026-05-18

**Authors:** Aakash Bavishi, Dean Marella, Jonathan H. Kim, Matthew W. Martinez

**Affiliations:** a Department of Medicine, Division of Cardiology, University of Illinois College of Medicine, Chicago, Illinois, USA; b Hypertrophic Cardiomyopathy, Atlantic Health System, Morristown Medical Center, Morristown, New Jersey, USA; c Emory Clinic Cardiovascular Research Institute, Emory School of Medicine, Atlanta, Georgia, USA; d Department of Cardiology, Mayo Clinic Foundation, Jacksonville, Florida, USA

**Keywords:** cardiomyopathy, echocardiography, imaging

## Abstract

**Background:**

Management of left ventricular outflow tract obstruction (LVOTO) in competitive athletes with hypertrophic cardiomyopathy (HCM) is challenging, particularly when obstruction is exercise-induced and medical therapy is poorly tolerated.

**Case Summary:**

A 28-year-old professional athlete presented with recurrent syncope. Resting imaging showed asymmetric septal hypertrophy without obstruction, but stress echocardiography revealed severe dynamic LVOTO (peak gradient: 150 mm Hg). Beta-blockers caused fatigue with persistent gradients. After shared decision-making, mavacamten was initiated. Follow-up stress imaging demonstrated complete resolution of LVOTO with preserved systolic function. The athlete returned to professional competition and remained event-free at 2 years.

**Discussion:**

Current guidelines emphasize shared decision-making for athletes with HCM. This case highlights the importance of stress imaging to unmask LVOTO and supports mavacamten as a viable alternative.

**Take-Home Messages:**

Stress testing is critical in symptomatic athletes with HCM. Mavacamten may permit alleviation of LVOTO and safe return to play.

## Visual Summary

**Figure:**
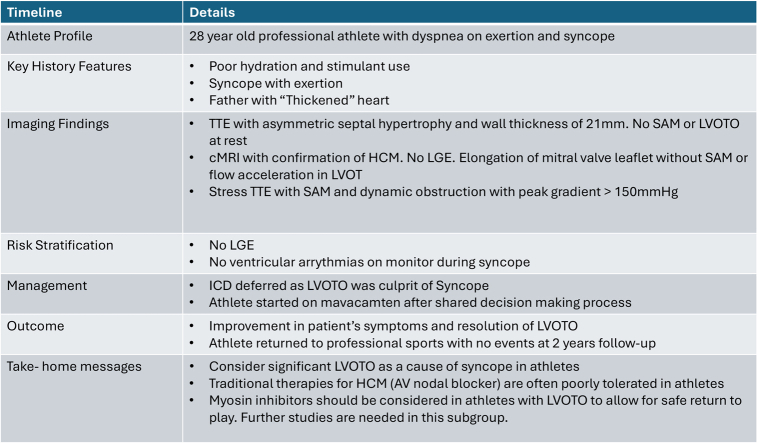

## History of Present Illness

A 28-year-old male professional athlete presented with exertional dyspnea and multiple episodes of syncope. After his first syncopal episode, he had an ambulatory monitor placed. He then had a second witnessed episode of syncope on the practice field, prompting immediate training staff evaluation. No cardiopulmonary resuscitation or defibrillation was required. The patient denied feeling palpitations or prodromal symptoms prior to syncopal episodes. His ambulatory monitor during the syncopal episode showed no ventricular arrythmias and only sinus tachycardia before syncope. He reported recent poor hydration and use of stimulants including Adderall and caffeine. In the cardiologist's office, his blood pressure was 118/73 mm Hg with a heart rate of 62 beats/min. Cardiovascular physical examination demonstrated regular heart rate and rhythm with no loud murmurs.

**Take-Home Messages**

• Management of LVOTO in elite athletes with HCM can be challenging given poor tolerance of atrioventricular nodal blocking agents and restrictions in return to competition after cardiac surgery.
• Mavacamten may be a safe and effective treatment for LVOTO in athletes. It is important that athletes with HCM continue to undergo annual risk stratification.

## Past Medical History

The patient had a history of attention deficit hyperactivity disorder. His family history was notable for his father having a “thickened heart.” He denied any family history of sudden cardiac death (SCD). He had no illicit substance use and did not drink alcohol.

## Differential Diagnosis

The differential diagnosis for this patient's syncope and exertional dyspnea included potential ischemic causes secondary to anomalous coronary artery or obstructive epicardial coronary artery disease. Potential arrhythmogenic causes included ventricular tachycardia secondary to underlying cardiomyopathies such as hypertrophic cardiomyopathy (HCM) or arrhythmogenic cardiomyopathy, long QTc syndrome, and other channelopathies. Vasovagal syncope could be considered; however, it would be unlikely given the syncope occurred during exercise.

## Investigations

**Figure 1:**
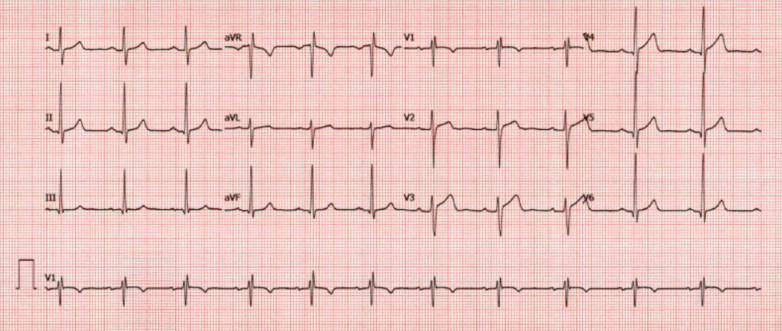
Athlete's Resting Electrocardiogram Electrocardiogram demonstrating normal sinus rhythm with normal voltages. There were no significant ST-T changes.

**Figure 2:**
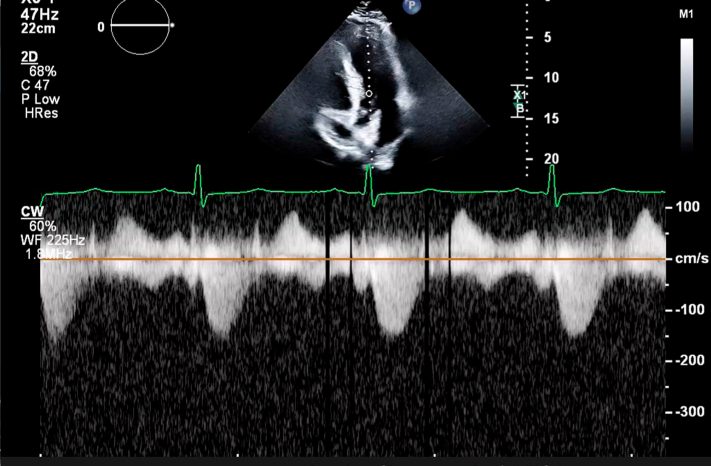
Athlete's Resting Left Ventricular Outflow Tract Gradient Continuous wave Doppler across the aortic valve displaying no significant left ventricular outflow tract gradient (<20 mm Hg).

The patient's electrocardiogram demonstrated sinus rhythm with normal voltages and no pathologic ST-T changes (Figure 1). Resting transthoracic echocardiography (TTE) (Video 1, Figure 2) demonstrated asymmetric septal hypertrophy with a maximal septal wall thickness of 21 mm. There was no evidence of systolic anterior motion (SAM) or left ventricular outflow tract obstruction (LVOTO) with Valsalva maneuvers (Video 1, Figure 2). Cardiac magnetic resonance imaging was performed for further assessment and demonstrated a maximal wall thickness of 22 mm without significant late gadolinium enhancement (LGE) (Video 2). The anterior leaflet was elongated, but there was no clear evidence of valvular SAM or flow acceleration in the left ventricular outflow tract (LVOT) (Video 3).

**Figure 3:**
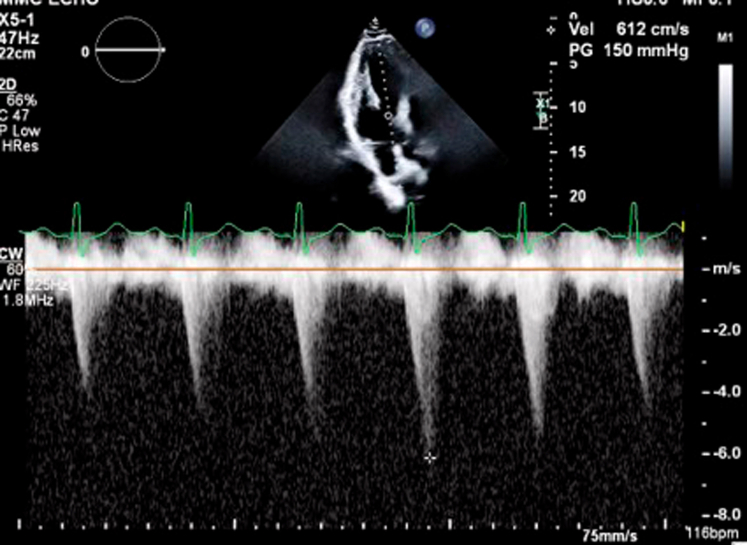
Athlete's Stress Left Ventricular Outflow Tract Gradient Continuous wave Doppler across the aortic valve during stress displaying late peaking dagger signal and significant left ventricular outflow tract obstruction with peak gradient near 150 mm Hg.

Stress TTE was pursued for evaluation of LVOTO with exercise. The patient exercised for over 16 minutes (18 METs), reaching stage 6 of the Bruce protocol with a normal heart rate and blood pressure response. He had no ventricular ectopy during stress testing. Immediate poststress images revealed significant SAM and color flow acceleration in the LVOT with severely elevated gradients (150 mm Hg), suggestive of dynamic obstruction (Video 4, Figure 3). The patient's 1-week Holter monitor (worn during syncopal episode) showed no ventricular arrythmias.

## Management (Medical/Interventions)

After multidisciplinary expert collaborations, placement of an implantable cardioverter-defibrillator was deferred for multiple reasons. The severe LVOTO—exacerbated by exertion, heat, poor hydration, and stimulant use—was presumed to be the culprit for the patient's syncope. The patient had no LGE, and no ventricular arrythmias were noted on Holter monitor, placing the patient at low risk for SCD.

To address LVOTO, the athlete was counseled on improving hydration and avoiding excess caffeine and limiting other stimulants. A low-dose beta-blocker was initiated, but he experienced fatigue with residual high LVOT gradients. An extensive shared decision-making conversation was performed with the athlete regarding next potential steps, including surgical myectomy versus myosin inhibitor. To avoid surgical myectomy per the athlete's preferences, mavacamten 5 mg was initiated.

## Outcome and Follow-Up

**Figure 4:**
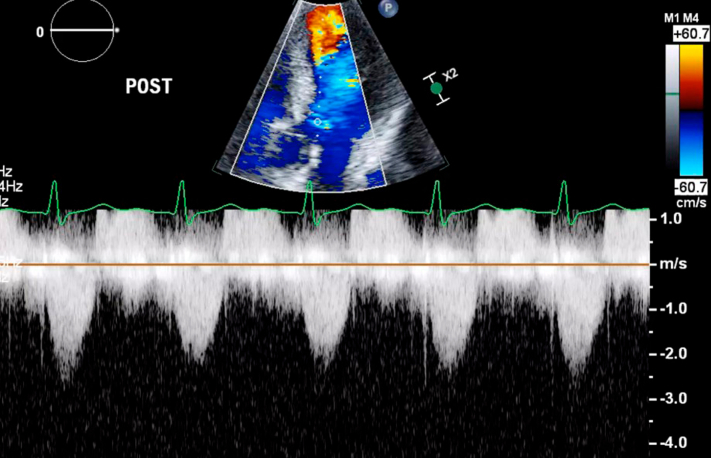
Athlete's Stress Left Ventricular Outflow Tract Gradient on 10 mg Mavacamten Continuous wave Doppler across the aortic valve displaying no significant left ventricular outflow tract gradient (<20 mm Hg).

The athlete responded well to mavacamten, with significant improvements in symptoms. Following appropriate Risk Evaluation and Mitigation Strategies (REMS) protocol, the mavacamten was uptitrated to 10 mg. Repeat stress echocardiography 3 months after initiation did not reveal any evidence of LVOTO with rest or stress (Video 5, Figure 4). The patient exercised for 18 minutes and reached 20 METs. His left ventricular ejection fraction remained normal with mavacamten.

The athlete was able to successfully resume professional sports and has not experienced any adverse cardiac events or further episodes of syncope over the past 2 years.

## Discussion

Recommendations for the management of HCM have dramatically changed over the past 20 years since the 2005 Bethesda Conference, where there was a Class 1A recommendation of competitive sports restriction in athletes with HCM.^1^ However, recent studies—including the LIVE-HCM study and a prospective study led by Martinez et al^2^ evaluating 40 elite athletes with HCM—have demonstrated the safety of vigorous activity and extremely low cardiac event rates with competitive sports in athletes with HCM.^3^ This has been reflected in the 2024 HCM and 2025 American College of Cardiology/American Heart Association sports cardiology guidelines, where there is now a Class 1 indication for athletes with HCM who wish to compete in sports to have a comprehensive evaluation and shared decision-making process with an expert professional.^4^^,^^5^ In stark contrast to 20 years ago, the guidelines now explicitly state that there is no benefit of universal restriction from competitive sports in athletes with HCM.^4^^,^^5^ It is critical that athletes with HCM undergo annual risk stratification for SCD with imaging and ambulatory monitors, as indicated.

As demonstrated in our case, in symptomatic patients it is critical that cardiologists evaluate for LVOTO. As was the case with our patient, stress TTE may be necessary to reproduce symptoms and elicit LVOTO. Use of stimulants, poor hydration, and competition in heat are all factors that can exacerbate LVOTO in athletes. It is essential that LVOTO is treated before an athlete is allowed to return to sports.

Treatment of LVOTO in athletes with HCM can be challenging. Previously, beta-blockers and nondihydropyridine calcium-channel blockers were mainstay treatments. However, these medications are commonly not options for athletes who rely on the ability to augment their heart rate and cardiac output at the highest level of competition. This is in addition to other unintended side effects or contraindications such as lethargy, fatigue, and baseline bradycardia and hypotension. Disopyramide, a third option, is commonly limited in its use owing to intolerable anticholinergic side effects.^6^ Septal reduction therapy via cardiac surgery would sideline an athlete for a prolonged period, with the likelihood of lifelong exclusion in some athletes.

This is to our knowledge the first case describing the use of mavacamten in a professional athlete. As demonstrated in the case, myosin inhibitors offer the potential to effectively and safely treat LVOTO in athletes and foster expedited return to play. Mavacamten is more likely to rapidly reduce LVOTO and should be better tolerated by elite athletes compared with high doses of atrioventricular nodal blockers. Head-to-head studies have demonstrated significantly worse peak Vo_2_ with beta-blockers compared with myosin inhibitors in HCM patients.^7^ The majority of HCM patients prefer myosin inhibitors compared with cardiac surgery, and it is likely that this would be especially true an in elite athlete population.^8^ Further studies are needed specific to mavacamten in an elite athletic population—including evaluating for incidence of atrial fibrillation—given that athletes have numerous hemodynamic and structural heart changes compared to the general population.^9^ Long-term data on the safety of myosin inhibitors exists up to 180 weeks, but studies with further-duration data are needed.^4^

## Conclusions

This case highlights several important principles in the management of an elite athlete with HCM: consideration of LVOTO as etiology of syncope, the utility of stress testing in eliciting LVOTO, the importance of annual risk stratification for SCD, and the utility of myosin inhibitors in treating LVOTO. This is the first reported use of mavacamten in an elite athlete to our knowledge. Further studies are needed to specifically study myosin inhibitors in an athletic population.

**Visual Summary:**
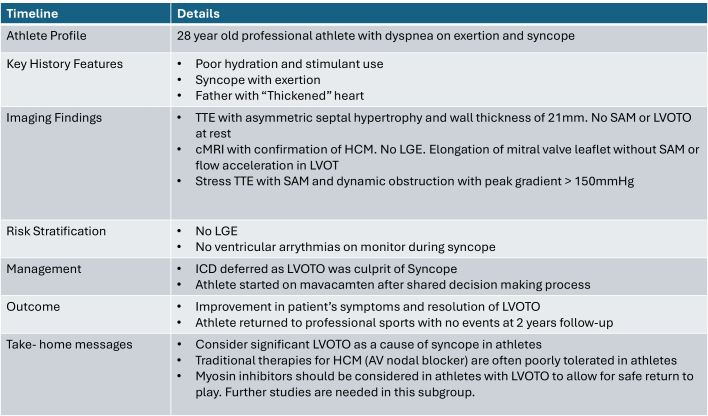
Athlete's Case Presentation A 28-year-old professional athlete with exertional syncope was found to have asymmetric septal hypertrophy without resting obstruction. Stress echocardiography revealed severe dynamic left ventricular outflow tract obstruction (>150 mm Hg). After intolerance to beta-blockers, mavacamten was initiated, with resolution of obstruction and symptoms, allowing return to professional sports without events at 2-year follow-up. AV = atrioventricular; cMRI = cardiac magnetic resonance imaging; HCM = hypertrophic cardiomyopathy; LVOT = left ventricular outflow tract; LVOTO = left ventricular outflow tract obstruction; ICD = implantable cardioverter-defibrillator; LGE = late gadolinium enhancement; SAM = systolic anterior motion; TTE = transthoracic echocardiography.

## Funding Support and Author Disclosures

The authors have reported that they have no relationships relevant to the contents of this paper to disclose.

## Appendix

Video 1 Athlete's Resting Transthoracic Echocardiogram Parasternal long-axis view demonstrates basal septal hypertrophy. Elongated anterior mitral valve leaflet but no clear evidence of SAM or dynamic obstruction.

Video 2 Athlete's Cardiac Magnetic Resonance Imaging Short-axis stack SSFP (steady-state free precession) cine images demonstrating basal septal hypertrophy up to 21 mm.

Video 3 Athlete's Cardiac Magnetic Resonance Imaging Gradient echo parasternal long-axis sequences demonstrating elongated anterior mitral valve leaflet with no clear evidence of SAM or flow acceleration in the LVOT.

Video 4 Athlete's Stress Transthoracic Echocardiogram Apical 3-chamber view demonstrates clear SAM and color flow acceleration in the LVOT.

Video 5 Athlete's Stress Transthoracic Echocardiogram on Mavacamten Apical 5-chamber view with no clear evidence of dynamic obstruction.
